# Spatial analysis of tuberculosis cure in primary care in Rio de Janeiro, Brazil

**DOI:** 10.1186/s12889-021-11834-1

**Published:** 2021-10-12

**Authors:** José Carlos Prado Junior, Roberto de Andrade Medronho

**Affiliations:** 1 Centro de Estudos Estratégicos, Fundação Oswaldo Cruz, Avenida Brasil 4036, 10° andar, Prédio da Expansão, Manguinhos, Rio de Janeiro, RJ 21040-361 Brazil; 2 Instituto de Estudos em Saúde Coletiva, Faculdade de Medicina, Universidade Federal do Rio de Janeiro, Rua Rodolpho Paulo Rocco, 255, 6° andar, Rio de Janeiro, RJ 21941-913 Brazil

**Keywords:** Family health, Infectious diseases, Primary care, Public health, Respiratory diseases, Tropical medicine

## Abstract

**Background:**

Tuberculosis (TB) presents a high burden of disease and is considered a global emergency by the World Health Organization (WHO), as the leading cause of death from infectious disease in adults. TB incidence is related directly to access to health services and socioeconomic determinants and inequality. Providing primary care settings can lead to improved access, shorter waiting times for patients, and enhanced TB case detection. The article aims to identify the spatial and temporal risk areas for TB and the relationship between TB cure and primary healthcare coverage from 2012 to 2014 in Rio de Janeiro, Brazil.

**Methods:**

A cross-sectional study was conducted in Rio de Janeiro, Brazil. All cases of TB reported to the Information System on Diseases of Notification (SINAN) from 2012 to 2014 were included. Socioeconomic variables from the 2010 Brazilian national census were also added. Socioeconomic variables were selected from multivariate analysis using principal factors analysis. Spatial association was verified with generalized additive model (GAM). It was possible to identify areas at higher risk of failure to cure TB.

**Results:**

TB rates showed strong positive spatial autocorrelation. TB cure rate varied according to schooling (individuals with complete secondary schooling had higher cure rates than illiterate individuals; OR 1.72, 95% CI 1.30–2.29), alcohol consumption (OR 0.47, 95% CI 0.35–0.64), contact investigation (OR 2.00, 95% CI 1.56–2.57), positive HIV serology (OR 0.31, 95% CI 0.23–0.42), and census tracts with higher elderly rates (OR 9.39, 95% CI 1.03–85.26). Individuals who had been covered by primary healthcare (PHC) for 35 to 41 months had 1.64 higher odds of cure, compared to those with no PHC coverage (95% CI 1.07–2.51).

**Conclusion:**

A comprehensive risk map was developed, allowing public health interventions. Spatial analysis allowed identifying areas with lower odds of TB cure in the city of Rio de Janeiro. TB cure was associated statistically with time of coverage by primary healthcare. TB cure rate also varied according to sociodemographic factors like schooling, alcohol abuse, and population density.

This methodology can be generalized to other areas and/or other public health problems.

**Highlights:**

- We studied standardized municipal TB cure rates in an area of social inequality in Brazil.
- TB rates showed strong positive spatial autocorrelation.
- Higher rates were associated with population density and socioeconomic conditions. Illiterate individuals were less likely to achieve TB cure.
- TB cure was less likely in individuals with HIV and alcohol abuse.
- TB cure was greater in areas with high primary healthcare coverage.

## Background

Tuberculosis (TB) remains the leading cause of death from infectious diseases worldwide, with 1.4 million deaths in 2019. Brazil is one of the 30 countries with highest TB burden, with an annual incidence rate of 46/100,000 inhabitants in 2019. Importantly, the country has a national vital statistics system and high-quality data [1].

In Brazil, all TB cases must be reported to the Information System on Diseases of Notification (SINAN; http:// portalsinan.saude.gov.br/), the national database for inclusion in the government database. The SINAN system, which commenced operation in 1997, stores details of all cases of selected diseases according to the list published by the Brazilian Ministry of Health. Case reports are transmitted to SINAN via standardized forms that include patient’s home address, clinical and laboratory data, and information on the treatment applied.

The most vulnerable people often bear a disproportionate burden of TB morbidity and mortality, with poverty, social vulnerability, and other social factors contributing to TB incidence [1–5]. Global TB prevalence is related to social inequality, poverty, overcrowding, migration, and inefficiency in TB control programs [6].

Although TB incidence and deaths have decreased in Brazil in recent years, the disease has increased in the vulnerable population, where diagnosis tends to be delayed and the odds of treatment dropout and death are significantly higher [7].

Simple policies can be delivered effectively in primary healthcare (PHC) to prevent TB in individuals at high risk [8, 9]. A consistent supply of primary care services can lead to improved access, better contact tracing, shorter waiting times for patients [10], and enhanced TB case detection [9].

Brazil has invested in mass social programs to improve health and equality, such as the Family Health Program [11], the gold standard model for PHC [12].

Rio de Janeiro, located in Southeast Brazil, presents a high TB incidence rate. The current TB control program in Rio de Janeiro is based on active case searches for patients exhibiting respiratory symptoms potentially associated with the disease.

In 2015, PHC coverage in the city of Rio de Janeiro was 46.16% [12, 13]. There were two PHC models: (1) Family Health Teams: general practitioners based on PHC principles (access, longitudinal care, coordination of care, and comprehensiveness); and (2) Traditional PHC: specialties based on gender and age (pediatrics, gynecology, clinicians) [12].

TB is known to bear a relationship to the territory [4–6]. Computer-based geographic information system (GIS) tools have become especially useful in TB control. They allow the detection of spatial clusters, and such information can be useful for understanding socioeconomic differences between neighborhoods, facilitating preventive measures, and developing more efficient and targeted policies for elimination of the disease, such as PHC coverage.

The current analysis addresses several methodological challenges associated with spatial modelling of TB cure and primary healthcare services.

First, although TB is the leading infectious cause of death worldwide, the numerical counts of TB cure are low in small areas, leading to instability in case counts and difficulty in distinguishing between true risk and risk from stochastic noise in individual geographic areas. A modelling approach that draws strength from the individual level across time and space could potentially stabilize these estimates.

Second, TB is associated with socioeconomic variables, but the latter are not available in the SINAN database. A modelling approach using population census data could allow making such estimates.

Furthermore, there are few studies that link primary healthcare coverage to TB cure. We can expect PHC to be associated with higher TB cure rates, but we do not know it takes (in months) following the deployment of a PHC team to produce better results with TB cure.

This article aims to identify spatial and temporal risk areas in relation to TB cure and the relationship to primary healthcare coverage from 2012 to 2014 in the city of Rio de Janeiro, Brazil.

## Methods

### Study design

This ecological study estimated the TB cure rate according to PHC coverage and socioeconomic, demographic, and epidemiological variables at the municipal level in Rio de Janeiro, Brazil, from 2012 to 2014.

### Setting

Rio de Janeiro is located in Southeast Brazil and is the second largest city in the country. According to the 2010 census conducted by the Brazilian Institute of Geography and Statistics (IBGE) [14], the population of Rio de Janeiro was estimated at 6,476,631 in 2015, distributed across 10,233 exclusively urban census tracts.

We chose the period from 2021 to 2014 because the PHC coverage in the city of Rio de Janeiro reached approximately 50% during this period, allowing better comparison of performance of care by services with versus without family health teams.

### Participants

The study included all incident cases of pulmonary and extrapulmonary TB reported to the SINAN database from 2012 to 2014.

Records from institutionalized patients were excluded from the study because it is impossible to define primary healthcare coverage for these cases.

Duplicate records and records without case conclusion were also excluded from the study.

All the sources were secondary data. None of the participants was contacted by the researchers.

### Data

Epidemiological and clinical data on cases were retrieved from the Brazilian National Information System on Diseases of Notification (SINAN) and geocoded according to patient’s residential address. Sociodemographic data were retrieved from the 2010 national census database.

Spatial distribution was observed to investigate the distribution of incident TB cases and possible areas with clusters of cases. The contribution of space and time to TB incidence adjusted by independent variables was addressed with the generalized additive model.

#### Vital data

Vital data included anonymized individual-level records from all new cases of pulmonary and extrapulmonary TB in Rio de Janeiro reported to SINAN from 2012 to 2014.

Data on gender, age, clinical form, and clinical evolution of the disease for each patient were retrieved from the SINAN database.

These records were tabulated by age group (0–25, 26–40, 41–75, and ≥ 75 years), sex (male; female), race/color (white; non-white), schooling (Illiterate, complete primary, complete secondary, university), and TB cure status.

#### Residential address geocoding

UTM coordinates (latitude and longitude) of the patient’s residence were determined from the home address recorded in the SINAN database using Google Maps Platform geocode tool [15].

Accuracy of the geocoding performed by Google Maps Platform can be assessed with a score ranging from 0 to 10 (0-not found, 1-country level, 2-state, 3-subregion, 4-city, 5-zip code, 6-streets, 7-street intersection, 8-address, 9-name of the building or business, 10-maximum precision). Addresses with scores less than “5” were considered losses. Acceptable accuracy was defined as scores from “8” to “10”. The remaining records were reviewed manually.

#### PHC coverage

PHC coverage in 2012, 2013 and 2014 was 38.9, 37.0, and 43.3% respectively, represented by geographic polygons.

A map was constructed using ARC-GIS® version 10.2.2 (Esri, Redlands, CA, USA) [16], by superimposing map points generated for TB cases geocoded at the household level on a digital map of PHC coverage marked with polygons in Latlong/WGS84 projection, available in the shapefile extension representing PHC coverage.

New TB cases were classified in two mutually exclusive categories according to PHC coverage. If the geocoded TB case matched the PHC coverage polygon, it was classified as “covered by PHC/Yes”. Otherwise, it was classified as “covered by PHC/No”.

Nearly all the PHC teams in Rio de Janeiro were created less then 3 years before 2012.

Therefore, to measure the time since creation of the PHC teams, we added a variable to the database for each record, called “PHC coverage time”. This variable was classified as “4 to 18 months”, “>18 to 35 months”, “>35 to 41 months”, and “> 41 months”.

#### Clinical and epidemiological covariates

The study’s dependent variable was “outcome, TB cure” (yes/no) obtained from SINAN-TB. The exposure variable was “PHC coverage time”, expressed as “time (in months) between implementation of the PHC team and TB diagnosis”.

To inform the model, we included covariates with a known or postulated epidemiological relationship with TB infection. The dimensions used were “environment”, “individual factors”, “access to healthcare services”, and “social status”. The model included demographic, social, and epidemiological variables and health services access and use.

The other variables selected from the SINAN-TB database were: age; gender; race/color (white/non-white); schooling; HIV coinfection (yes/no); history of alcohol abuse (yes/no); contact search (yes/no); HIV serology (positive, negative, not performed); and supervised TB treatment (yes/no).

#### Sociodemographic covariates

We included sociodemographic information from the population census by the Brazilian Institute of Geography and Statistics (IBGE) [17]. These records were tabulated at the census tract level.

There is a large number of socioeconomic variables from the 2010 population census [17]. Multivariate analysis was performed using principal components analysis (PCA) to identify small numbers of principle components that explain most of the variation in a dataset [18].

Socioeconomic and demographic covariates included (i) “head-of-household’s mean monthly income (Brazilian *reais*)”; (ii) “mean number of residents per household”; (iii) “population density in the census tract”; (iv) “density of residents per room”; (v) “proportion of permanent private households with bathrooms for exclusive use of residents or bathrooms and sewage disposal via sewage system or storm drains”; (vi) “proportion of permanent households with electricity”; (vii) “average number of bathrooms per permanent private residence”; and (viii) “elderly rate”.

These mean socioeconomic and demographic values were repeated for each individual resident in the same census tract since these variables at this level of aggregation showed high homogeneity. The other variables were analyzed at the individual level.

### Bias

Selection bias can occur in a secondary data source. To minimize this bias, we attempted to include all patient records of new TB cases. To avoid classification bias in terms of primary healthcare coverage, institutionalized patients were excluded from the study.

A known classification bias in studies that use geocoding coordinates from addresses is the failure to identify coordinates precisely. This may compromise the classification of PHC coverage rating. To minimize this bias and improve the completeness and assertiveness of geocoding, a manual review of all results with low precision was performed, and those in which it was not possible to complete the definition of the coordinates for the purposes of spatial analysis were considered losses to the study.

### Statistical analysis

#### Descriptive analysis

Statistical analysis was performed with the R software (version 4.0.3. for MacOs) [19]. The study employed descriptive statistics to examine the explanatory variables. The independent variables selected were submitted to exploratory analysis, using frequency tables, boxplots, and histograms.

A univariate generalized linear model (GLM) was used to examine the association between the dependent variable TB cure and the independent variables. GLM models were introduced by Nelder et al. (1989) [20] as an extension of linear models. GLM is a unified theory of linear models for categorical or continuous variable responses.

Calculation of the odds ratio for each variable was performed. Statistical significance was set at alpha of 0.05.

In GLM, the expected value of the distribution of the variable response Y_i_ is modeled. A generalized linear model has two components: random and systematic. The random component is the variable response that must belong to the exponential family.

The systematic component describes the relationship between the covariates through the linear predictor η_i_:

$$ {n}_i=\beta^{\prime }{x}_i $$

Where:

*x*_*i*_ = explanatory variable vector.

*β*′ = coefficient vector of covariates.

The connection between the systemic components and the expected value of the variable response (*μ*_*i*_) is made through a function of g (.) Connection function:

$$ g\left({\mu}_i\right)={n}_i $$

The estimation process is performed using iterative methods of weighted least squares.

After adjusting the models, it is necessary to choose the best fit, for which the AIC (Akaike information criterion) is widely used in the comparison between nested and non-nested models. The basic idea is to select a model that is parsimonious, that is, with a reduced number of parameters to be estimated, but with a good fit. This criterion, which is based on the likelihood function (L) and penalizes the number of parameters (p), is given by

$$ AIC=-2 logL+2p $$

For models with the same dataset, the best model is the one with the lowest AIC value [21].

### Spatial analysis

Exploratory spatial analysis is a powerful instrument in spatial health research by virtue of its capacity to map disease distribution and associated risk factors at the population level.

#### Density estimation

For the spatial exploratory analysis, we used the geocoded map performed with the residential addresses of new TB cases.

A smooth kernel density map of TB cases map was employed in the visual identification of areas exhibiting the highest numbers of cases/m^2^ of surface. This statistical smoothing technique allowed filtering for the variability of the dataset while retaining the essential characteristics of the data locations [22]. This technique consists of generating a point density surface within a region of influence, weighted by the distance of each from the location of interest, for the visual identification of “hot areas” on the map. It is widely used for visualizing distribution patterns of point data.

In kernel density analysis, the visualized data can change according to how the spatial bandwidth is determined and which function is selected. In this study, spatial bandwidth was calculated as follows:

$$ \mathrm{SearchRadius}=0.9\times \min \left( SD,\sqrt{\frac{1}{\ln (2)}}\times {D}_m\right)\times {n}^{-0.2} $$

Where:

SD = standard distance.

*D*_*m*_ = mean distance.

n = sum of population field values.

Bandwidths from 500 m to 3000 m were tested, with 250 m increments.

According to our calculation, the spatial bandwidth was set to 2500 m because it was considered the most appropriate for highlighting strategic areas. We generated maps with estimates of TB incidence rate through the kernel ratio between reported TB cases and the population’s kernel.

#### GAM model

Spatial analysis was based on the generalized additive model (GAM), which can be considered an extension of generalized linear models, with the inclusion of a nonparametric element by smoothing functions.

The linear predictor predicts some known smooth monotonic function of the expected value of the response, and the response may follow any exponential family distribution, or simply have a known mean variance relationship, permitting the use of a quasi-likelihood approach.

The model has the great advantage of being more flexible and relatively simple to interpret [23, 24].

In general, the model has a structure:

$$ \mathrm{g}\left({\mu}_i\right)={A}_i\theta +{f}_1\left({x}_{1i}\right)+{f}_2\left({x}_{2i}\right)+{f}_3\left({x}_{3i},{x}_{4i}\right)+\dots $$

Where:

*μ*_*i*_ ≡ Ε(*Y*_*i*_) and *Y*_*i*_~*EF*(*μ*_*i*_, ∅.)

*Y*_*i*_ = response variable

*EF*(*μ*_*i*_, ∅) = exponential family distribution with mean μi and scale parameter, φ

Ai is a row of the model matrix for any strictly parametric model components

θ is the corresponding parameter vector

fj are smooth functions of the covariates, xk.

The method allows generating a risk surface that identifies areas of risk or protection, controlled by individual variables.

Variables with smoothing indicated in the model with the s () function can be plotted separately, and the point data can be plotted on a layer similar to that of a kernel.

In its estimation, the link function, g (μi), is replaced, where g (μi) = ΣXß, where X represents the vector of the explanatory variables and ß the vector of parameters to be estimated for g (μi) = Σ f (X), where f (x) is a nonparametric function.

In general, its structure resembles:

$$ E(Y)=f\left({X}_1\dots, {X}_n\right)={\beta}_0+{\beta}_1\left({X}_1\right)+\dots ++{\beta}_n\left({X}_n\right)+g(s)+\varepsilon $$

Where:

*g*(*s*) = represents a smoothed function of (x, y), which results in the estimation of a spatial surface

There is more than one technique available to generate the smoothed surface. Among them, the most commonly applied are based on splines, as they provide intuitively pleasant functions. Thin plate regression splines over location were used to account for autocorrelation [25]. Created in 1977 by Duchon, they provide an elegant way to estimate the smoothing function in a multivariate model with possible noise in the observations. For this option, it is not mandatory to choose nodes (or possible break numbers), which if done manually can be an extremely subjective task. This function was built as almost an ideal straightener, as it specifically defines how much weight to assign and because it is objective [26].

#### Analytical model

**Fig. 1:**
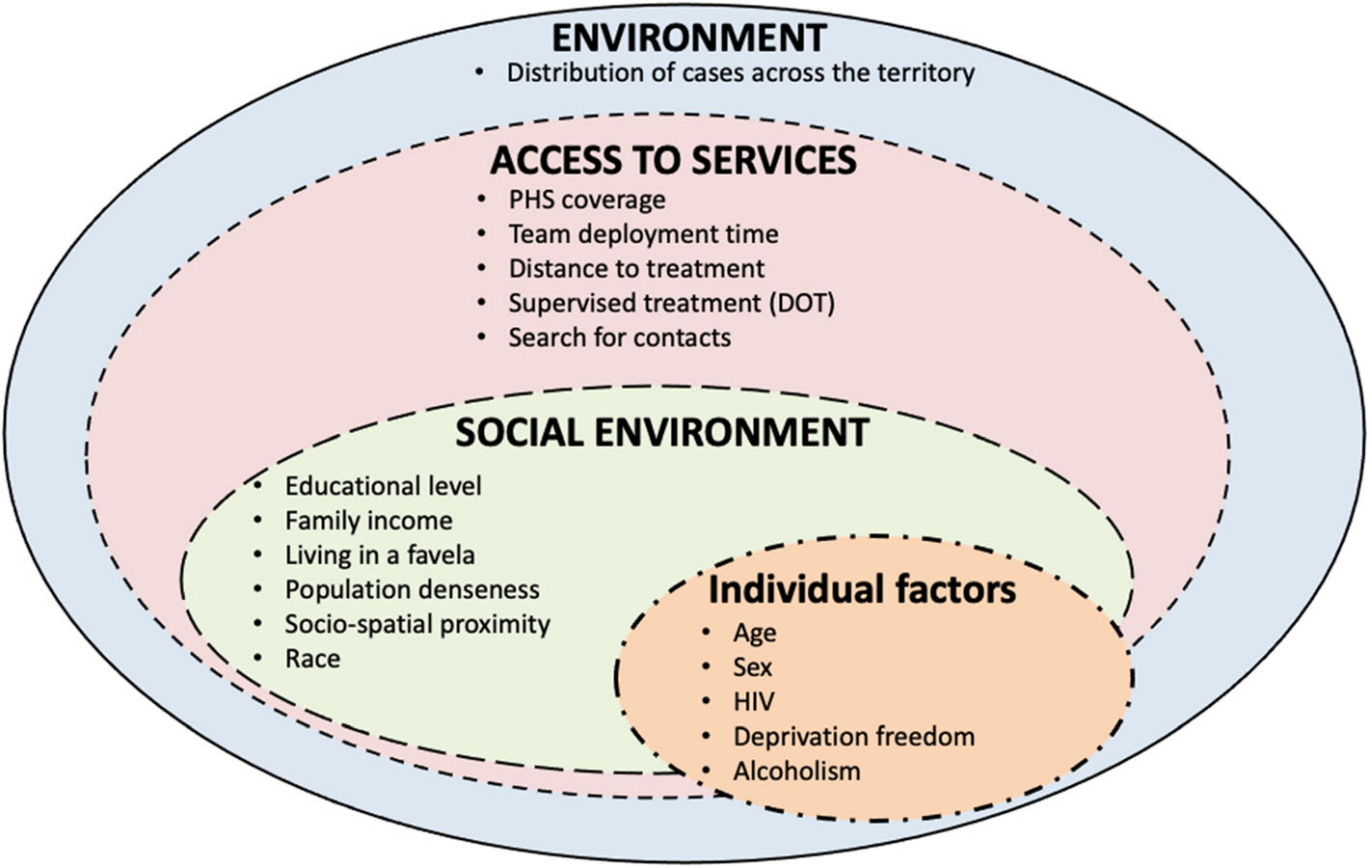
Generic Theoretical Model for TB cure

For all health problems, it is intuitive to imagine that there are more distal factors that are barely measurable, but that influence the pattern of illness in a population. For this reason, a more generic design was started that guided the analyses (Fig. 1). To cure tuberculosis, an individual with the disease must have been diagnosed and received treatment. The environmental, individual, and health services access factors and social conditions (crowding, income, schooling, household conditions, and demographic factors) of the individual influence the transmission, diagnosis, and treatment of tuberculosis.

Construction of the generalized additive models was performed through manual selection, based on the essential factors of the theoretical framework, not only considering the statistically significant variables in the bivariate analysis, but also those of epidemiological importance.

We considered not only the *p*-value of each association, but each variable’s previously described importance and the impact on the model’s explanatory power. Only those variables with a clear negative impact on the model’s explanatory power, observed through deviance, were removed.

In this study we performed the following model:

$$ \mathit{\log}\left\{\frac{p\left(s,x\right)}{1-p\left(s,x\right)}\right\}={\beta}_0+{\beta}_{1s}\left( Xcoord, Ycoord\right)+{\beta}_2\left( Tbcure==2\right)+{\beta}_3(age)+{\beta}_4\left( income<2\right)+{\beta}_5\left( pop\  density>20\right)+{\beta}_6\left( sewage<2\right)+{\beta}_7\left( elderly\ rate>0.2\right)+\varepsilon $$

Model analyses were conducted in the R software, version 4.0.3 [19] using distinct packages.

The MASS [27] and car [28] packages were used for the Poisson and negative binomial regression. Generalized additive models were run using mgcv [23]. Package descr [29] was used for weighted frequency and contingency tables of categorical variables and for comparison of the mean value of a numerical variable by levels of a factor. Classes and methods for spatial data were used with the sp. [30, 31], sdep [31, 32], maptools [33] R packages. Utility functions are provided, e.g., for plotting data as maps, spatial selection, as well as methods for retrieving coordinates, for sub-setting, print, and summary.

The Splancs [34] package was used for display and analysis of spatial and time point pattern analysis.

For curve, surface, and function fitting with an emphasis on splines, spatial data, geostatistics, and spatial statistics, we used the fields [35] package.

During the analyses, the following additional packages of R were used: dplyr [36] for working with data frame-like objects, tidyverse [37] for data science tools, RColorBrewer [38] for color schemes for maps, and ggplot2 [39] for creating graphics.

This study complies with the Guidelines for Accurate and Transparent Health Estimates Reporting (GATHER; http://gather-statement.org). Analyses were done with R version 4.0.3 [19]. This complies also with Resolution 466/12 of the Brazilian National Health Council. The study was submitted to and approved by the Institutional Review Board of the Rio de Janeiro Municipal Health Department.

## Results

### Loss rate

In the study, 15,458 new cases were closed in the years 2012 to 2014 in the city of Rio de Janeiro. Of these, 1074 cases were excluded from the analysis because they were institutionalized patients, for whom it is not possible to define the coverage of primary healthcare.

Of the remaining 14,384 records, we reached 10,900 georeferenced records. Therefore, the loss rate was 24.22% (3484 of 14,384 records).

### TB epidemiological findings

The cure rate was 71.57% (11,063 new cases) of the total cases closed. The mean incidence rate was 84.91 cases/100,000 inhabitants. In the same period, there were 725 cases of death by TB resulting in a mean specific mortality rate of 3.76 deaths per 100,000 inhabitants, and the case-fatality rate was 4.69% among new cases closed. Median age was 38 years.

### Density estimates

**Fig. 2:**
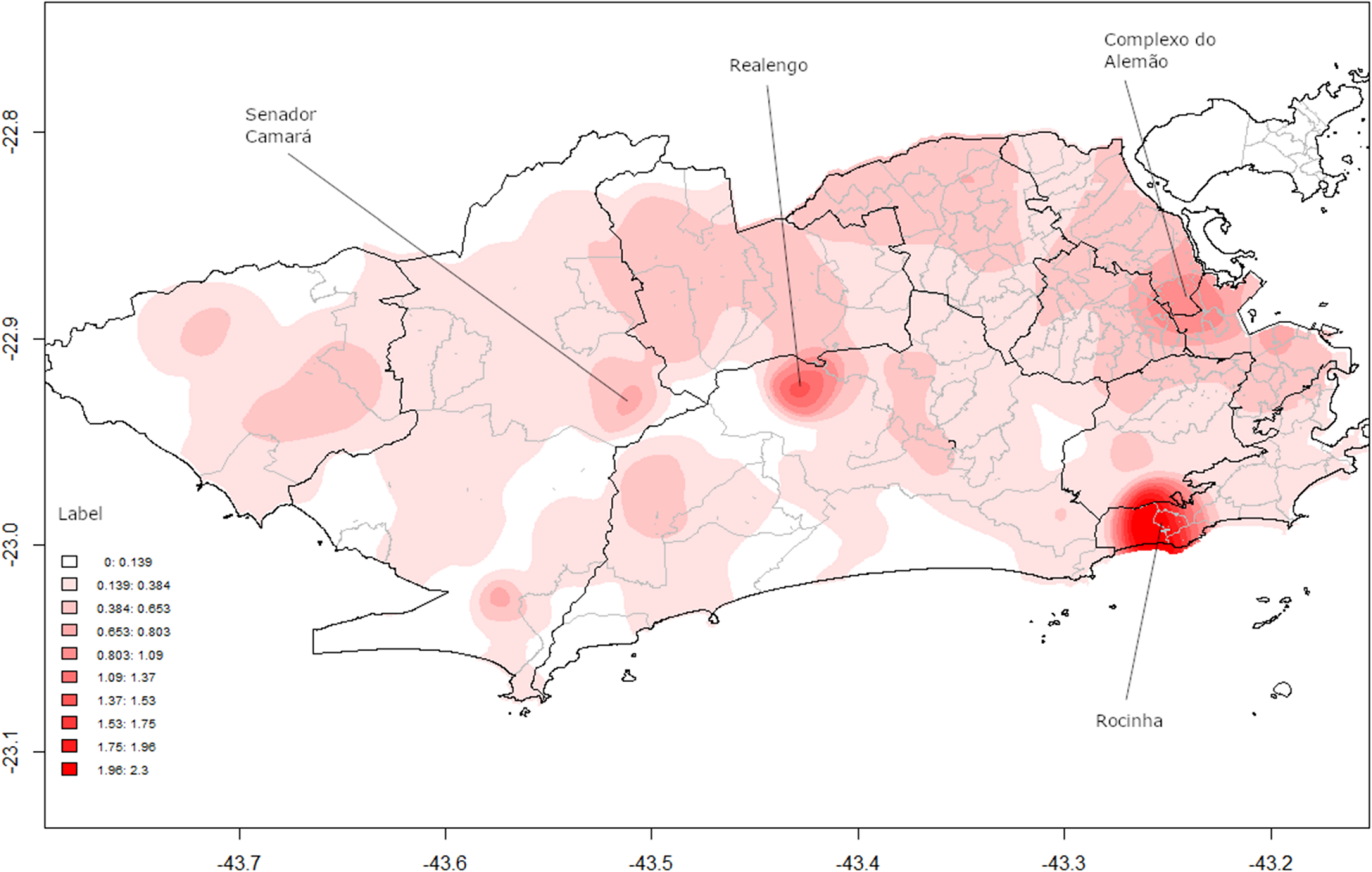
Kernel ratio between new TB cases and population. City of Rio de Janeiro (RJ), Brazil, 2012–2014. Source: Cartographic data: IPP, Epidemiological data: SINAN-TB. Projection GCS. Datum WGS84

Estimation of the incidence rate from the kernel ratio shows that the “hot areas” for new TB cases were mainly concentrated in the South Zone (Rocinha), followed by the North Zone (Complexo do Alemão, Acari, Pavuna) and West Zone (Senador Camará, Realengo) (Fig. 2).

### Analysis of TB cure and socioeconomic variables

Most TB cases occurred in males (*n* = 8919; 62.0%), non-white individuals (*n* = 7839, 54.5%), and illiterates (*n* = 4493, 31.2%).

An association was found (Table 1) in the crude bivariate analysis between TB cure and socioeconomic, demographic, and epidemiological variables. In relation to age, there was a lower probability of cure in the age group “> 25 to 50 years” (OR 0.86, 95% CI 0.78–0.94) compared to the reference category (“0 to 25 years”). Among individuals aged “> 80 years”, the odds of cure decreased even further (OR 0.44, 95% CI 0.32–0.59). Women were 1.51 times (95% CI 1.39–1.63) more likely to be cured than men. Non-white individuals were less likely to be cured (OR 0.68, 95% CI 0.62–0.73) than whites. Individuals with more schooling were more likely to be cured, compared to illiterate persons.

**Table 1:** Crude cure analysis of new TB cases and socioeconomic variables (*N* = 14,384). City of Rio de Janeiro (RJ), Brazil, 2012–2014

Variables (*N* = 14,384)	Cure						OR	95% CI	*p*-value
	Yes		No		Total				
	n	%	n	%	n	%			
Age									
0 to 25 years	2759	26.2%	926	24.0%	3685	25.6%	1.00		
> 25 to 50 years	4865	46.2%	1908	49.5%	6773	47.1%	0.86	0.78–0.94	< 0.001^b^
> 50 to 80 years	2745	26.1%	923	23.9%	3668	25.5%	1.00	0.9–1.11	0.975
> 80 years	96	0.9%	74	1.9%	170	1.2%	0.44	0.32–0.59	< 0.001^b^
Missing	65	0.6%	23	0.6%	88	0.6%	0.95	0.59–1.53	0.839
Sex									
Male	6264	59.5%	2655	68.9%	8919	62.0%	1.00		
Female	4264	40.5%	1199	31.1%	5463	38.0%	1.51	1.39–1.63	< 0.001^b^
Race/color									
White	4243	40.3%	1169	30.3%	5412	37.6%	1.00^a^		
Non-white	5570	52.9%	2269	58.9%	7839	54.5%	0.68	0.62–0.73	< 0.001^b^
Missing	717	6.8%	416	10.8%	1133	7.9%	0.47	0.41–0.54	< 0.001^b^
Schooling									
Illiterate	3186	30.3%	1307	33.9%	4493	31.2%	1.00^a^		
Complete primary	1747	16.6%	496	12.9%	2243	15.6%	1.44	1.28–1.63	< 0.001^b^
Complete secondary	1923	18.3%	389	10.1%	2312	16.1%	2.03	1.79–2.3	< 0.001^b^
University	619	5.9%	100	2.6%	719	5.0%	2.54	2.04–3.17	< 0.001^b^
Missing	3055	29.0%	1562	40.5%	4617	32.1%	0.80	0.73–0.88	< 0.001^b^
Head-of-household’s mean monthly income (BRL)							1.001	1.001–1.004	< 0.001^b^
Mean number of residents in permanent private households							0.77	0.69–0.85	< 0.001^b^
% permanent households with electricity							2.16	0.57–8.06	< 0.001^b^
Mean number of bathrooms per household							1.33	1.20–1.48	< 0.001^b^
Elderly rate							5.87	2.77–12.43	< 0.001^b^

SOURCE: Municipal Databases, SINAN (DATASUS/MS) and IBGE, 2010. Updated 10 January 2016

*Abbreviations*: *n* sampling size, *OR* odds ratio, *95% CI* 95% confidence interval, *p*-value - significance level

^a^ reference category; ^b^ statistical significance *p* < 0.05

The socioeconomic variables revealed an association between TB cure and mean income with OR 1.001 (95% CI: 1.001–1.004), mean number of residents in permanent private households OR 0.77 (95% CI: 0.69–0.85), percentage of permanent households with electricity OR 2.16 (95% CI: 0.57–8.06), mean number of bathrooms per household OR 1.33 (95% CI: 1.20–1.48), and elderly rate OR 5.87 (95% CI: 2.77–12.43).

### Analysis of TB cure and epidemiological and healthcare variables

Individuals without AIDS coinfection were 3.46 (95% CI: 3.07–3.91) times more likely to be cured of TB when compared to coinfected individuals (Table 2). Likewise, individuals with negative HIV serology were 3.67 (95% CI 3.28–4.12) times more likely to be cured than those with positive HIV serology. Meanwhile, individuals with unsupervised treatment were less likely to be cured (OR 0.65, 95% CI 0.60–0.70) compared to those whose treatment was supervised, and cases with no contact tracing (household contact investigation) were also less likely to be cured (OR 0.36, 95% CI 0.33–0.39) than those with contact investigation. As for primary healthcare (PHC) coverage, a slightly higher likelihood of cure was seen with higher PHC coverage, but without statistical significance.

**Table 2:** Crude analysis of cure of new TB cases and epidemiological and healthcare variables (*N* = 14,384). City of Rio de Janeiro (RJ), Brazil, 2012–2014

Variables (*N* = 14,384)	Cure						OR	95% CI	*p*-value
	Yes		No		Total				
	n	%	n	%	n	%			
HIV coinfection									
Yes	606	8.0%	643	23.1%	1249	12.1%	1.00		
No	6.977	92.0%	2139	76.9%	9116	87.9%	3.46	3.07–3.91	< 0.001^b^
HIV test performed									
Positive	744	7.1%	707	18.3%	1451	10.1%	1.00		
Negative	7.573	71.9%	1960	50.9%	9533	66.3%	3.67	3.28–4.12	< 0.001^b^
Not performed	2.213	21.0%	1187	30.8%	3400	23.6%	1.77	1.56–2.01	< 0.001^b^
Supervised TB treatment									
Yes	6.181	65.2%	1895	54.9%	8076	62.4%	1.00		
No	3.300	34.8%	1556	45.1%	4856	37.6%	0.65	0.6–0.7	< 0.001^b^
Contact investigation									
Yes	8.572	81.4%	2359	61.2%	10,931	76.0%	1.00		
No	1.958	18.6%	1495	38.8%	3453	24.0%	0.36	0.33–0.39	< 0.001^b^
Alcohol abuse									
Yes	769	8.7%	524	17.2%	1293	10.9%	1.00		
No	8.066	91.3%	2517	82.8%	10,583	89.1%	2.18	1.94–2.46	< 0.001^b^
Time covered by PHC									
4 to 18 months	1.211	26.8%	417	25.8%	1628	26.6%	1.00		
> 18 to 35 months	1.766	39.1%	680	42.0%	2446	39.9%	0.89	0.78–1.03	0.115
> 35 to 41 months	418	9.3%	124	7.7%	542	8.8%	1.16	0.92–1.46	0.207
> 41 months	1.118	24.8%	397	24.5%	1515	24.7%	0.97	0.83–1.14	0.717

SOURCE: Municipal databases, SINAN (DATASUS/MS) and IBGE, 2010. Updated 10 January 2016

*Abbreviations*: *n* sampling size, *OR* odds ratio, *95% CI* 95% confidence interval; *p*-value - significance level

^a^ reference category; ^b^ statistical significance *p* < 0.05

### Spatial analysis

**Fig. 3:**
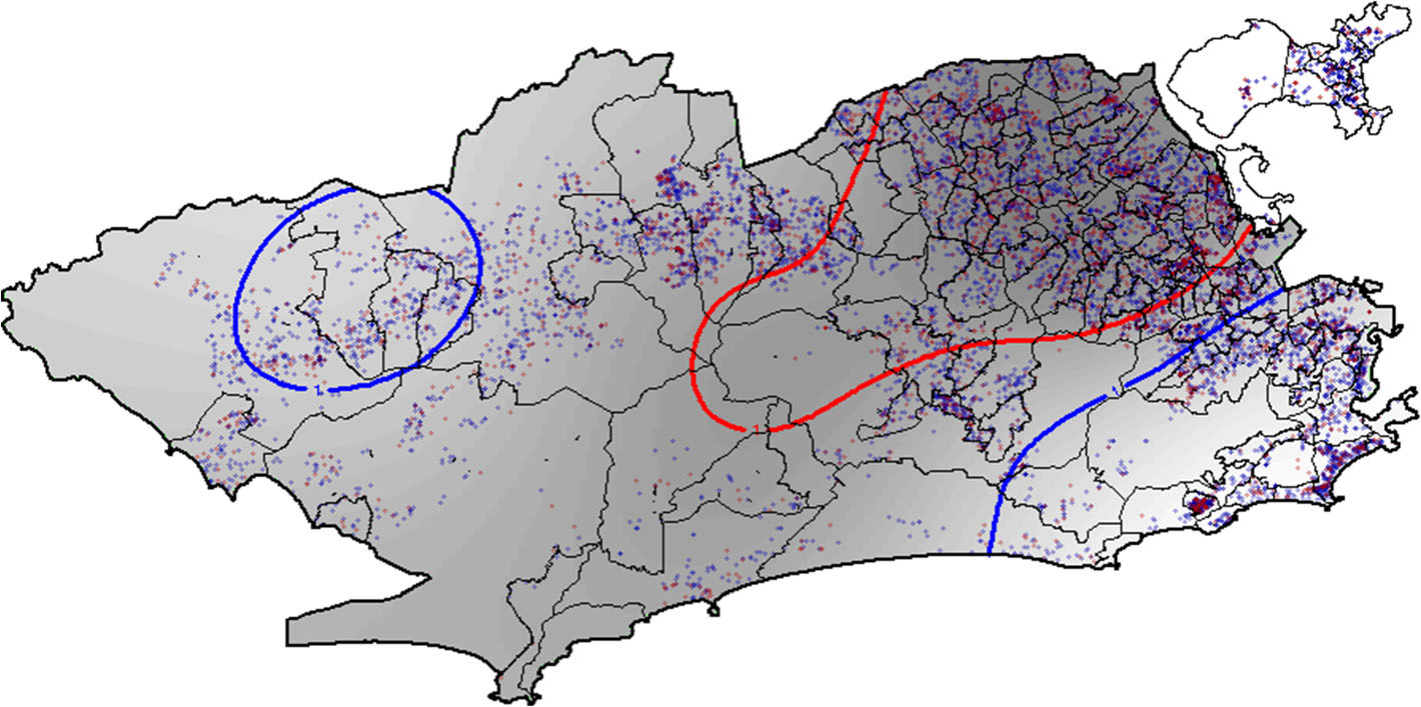
Probability map of TB cure using the generalized additive model (GAM) for cases (not cured - red) and controls (cured - blue). Rio de Janeiro, 2012 to 2014. Source: Cartographic data: IPP, Epidemiological data: SINAN-TB. Projection GCS. Datum WGS84

The probability map for TB cure was developed with the spatial generalized additive model (GAM). Thus, it was assumed that “cases” were all TB cases without cure as the outcome (lack of cure), while “controls” were recorded with cure as the outcome (Fig. 3).

Individuals residing within the contours in the West and South Zones (upper left and lower right sides of the map) represent areas with OR significantly greater than 1. Individuals residing in those areas tend to have higher TB cure rates, whereas those living in the North Zone, within the contour (upper central contour of the map) tend to have less likelihood of cure.

Table 3 shows the final model of the spatial analysis of TB cure and socioeconomic, demographic, and epidemiological variables using the generalized additive model.

**Table 3:** Final model of spatial analysis of TB cure for new TB cases and socioeconomic, demographic, and epidemiological variables using the generalized additive model - GAM (*N* = 14,384). City of Rio de Janeiro (RJ), Brazil, 2012–2014

Variable	Coefficient	Error	OR	95% CI	***p***-value
PHC coverage 4 to 18 months	0.3101913	0.1337	1.36	1.04–1.77	0.020*
PHC coverage >35 months to 41 months	0.4972078	0.2178	1.64	1.07–2.51	0.022*
PHC coverage > 41 months	0.0982589	0.1378	1.10	0.84–1.44	0.476
Alcohol abuse (yes)	−0.7423219	0.1556	0.47	0.35–0.64	< 0,001*
Complete Primary Schooling	0.1327906	0.1345	1.14	0.87–1.48	0.323
Complete Secondary Schooling	0.5474371	0.1454	1.72	1.30–2.29	< 0,001*
University	0.4809512	0.2641	1.61	0.96–2.71	0.068.
Contact Search (yes)	0.6972460	0.1273	2.00	1.56–2.57	< 0,001*
Positive HIV serology (yes)	−1.1426839	0.1470	0.31	0.23–0.42	< 0,001*
Supervised treatment (yes)	0.1781498	0.1271	1.19	0.93–1.53	0.160
Age group 0 to 25 years	0.0005724	0.1332	1.00	0.77–1.29	0.996
Age group > 50 to 80 years	0.3062125	0.1339	1.35	1.04–1.76	0.022*
Age group > 80 years	−1.1596947	0.5508	0.31	0.10–0.92	0.035*
Elderly rate	2.2402193	1.1253	9.39	1.03–85.26	0.046*

SOURCE: Municipal databases, SINAN (DATASUS/MS) and IBGE, 2010. Updated 10 January 2016

Deviance: 9.16%; AIC/UBRE: −0.049082

*Abbreviations*: *OR* odds ratio, *95% CI* 95% confidence interval; *p*-value - significance level

^*^ Statistical significance *p* < 0.05

**Fig. 4:**
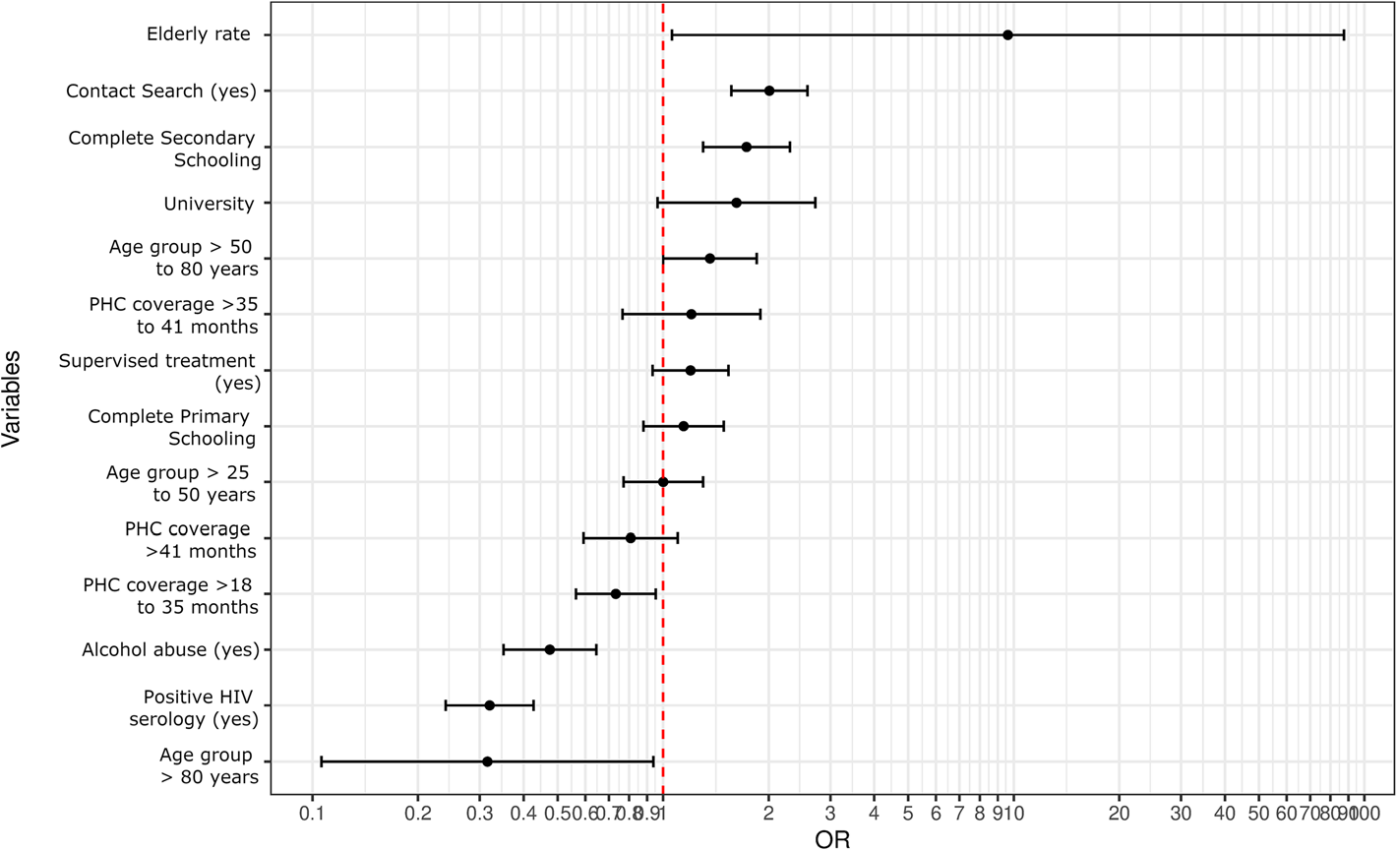
Final spatial analysis model using the generalized additive model (GAM) for TB cure. Rio de Janeiro, 2012 to 2014

Table 3 shows that PHC coverage, measured here as time between implementation of PHC and diagnosis, was significant for the categories “4 to 18 months” and “> 35 months to 41 months”, with OR 1.36 (95% CI 1.04–1.77) and OR 1.64 (95% CI 1.07–2.51) respectively. Individuals with a history of alcohol abuse had about half the likelihood of cure (OR 0.47, 95% CI 0.35–0.64), compared to individuals with no history of alcohol abuse. There was a positive trend in the association between schooling and odds of TB cure, especially in the case of secondary schooling, with 1.72 odds of cure (95% CI 1.30–2.29) compared to illiterate individuals. Individuals with positive HIV serology were less likely to achieve TB cure (OR 0.31; 95% CI 0.23–0.42). Meanwhile, individuals residing in census tracts with higher elderly rates were 9.39 (95% CI 1.03–85.26) times more likely to achieve cure. Figure 4 graphically shows the odds ratio values.

**Fig. 5:**
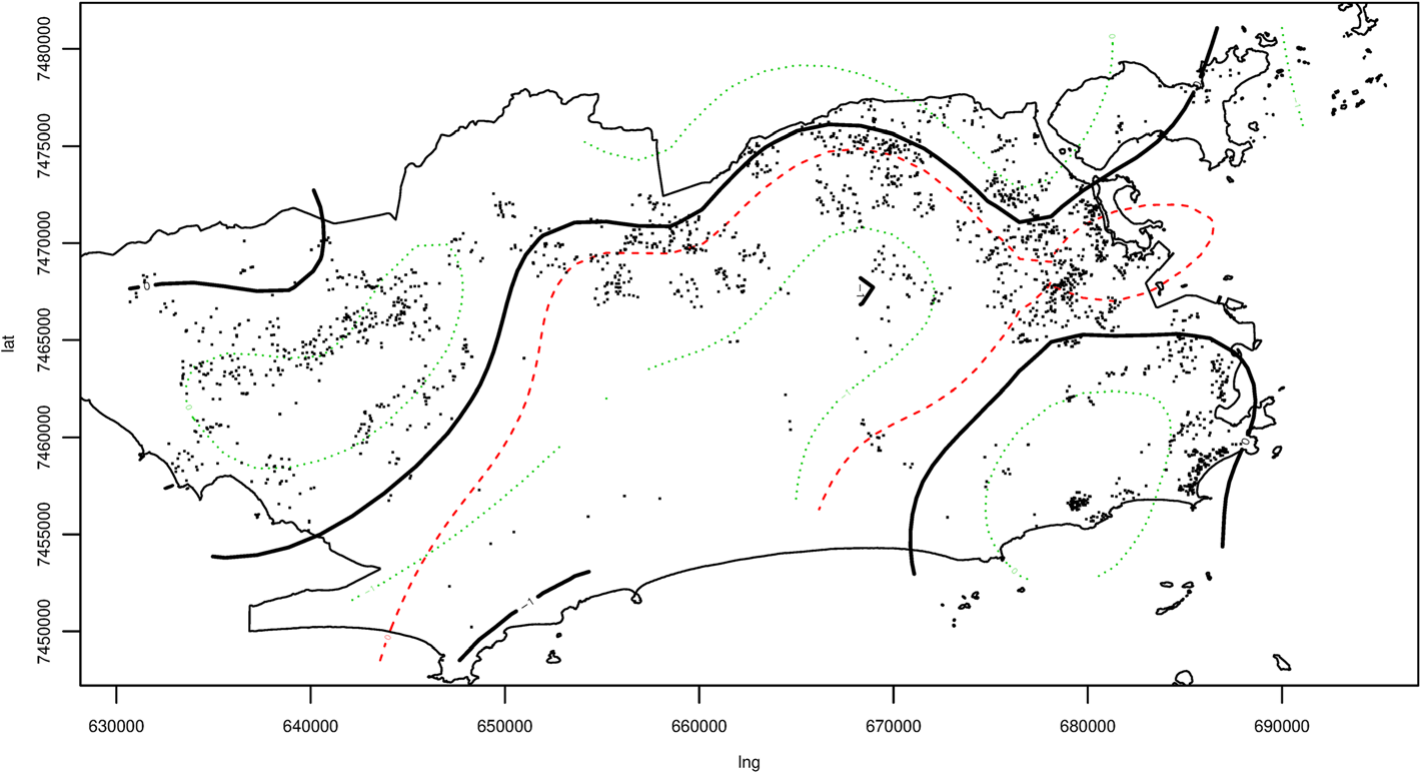
Final spatial analysis model using the generalized additive model (GAM) for TB cure - smoothed spatial component adjusted for the other covariates. Rio de Janeiro, 2012 to 2014

Figure 5 shows the map of the final spatial model. It is the smoothed spatial component, adjusted for the final model’s other socioeconomic, demographic, and epidemiological variables. Significant spatial association was found (*p* = 0.0219). The areas surrounded by green dotted lines had positive spatial correlation for TB cure, whereas red dotted lines had inverse spatial correlation with cure.

## Discussion

### Principal findings

We studied standardized municipal TB cure rates in an area of urban social inequality in Brazil.

Few studies in Brazil have addressed the spatial distribution of endemic diseases such as TB in urban areas. Information on the spatial and temporal spread of these diseases allows understanding the occurrence of these events in the territory. In addition, the description and visualization of the events´ spatial distribution facilitate the identification of their association with local characteristics such as socioeconomic conditions.

Tuberculosis rates showed strong positive spatial autocorrelation and association with socioeconomic and demographic variables.

Mapping of cases is a convenient tool for spatial characterization of TB, the results of which promote a better understanding of TB distribution and of the areas with greatest risk of infection.

The results showed that primary healthcare coverage was related to higher TB cure rates, besides better contact tracing and higher rates of supervised treatment. The authors believe that PHC allows achieving shorter waiting times for treatment and better access to health services and TB preventive measures.

### Comparison with other studies

The TB cure rate of 71.57% was lower than in other studies conducted in other regions of Brazil, such as 90.9% TB cure in the state of Maranhão [40]. However, it is higher than in other studies; Lima et al. (2020) [41] found a median cure rate of 29.8% among cities in Northeast Brazil. Two other studies identified decreases in TB cure rates in some cities in the state of Sergipe [4] and in Fortaleza, Ceará [5], in recent years.

Spatial analysis showed a significant spatial association with TB cure. In addition, the TB cure probability map shows that patients in the South and West Zones of Rio de Janeiro were more likely to achieve TB cure, while those in the North Zone were less likely to achieve cure. This result can be useful for public health policy purposes since it is possible to prioritize this region to improve TB cure in the city. The regions with the highest likelihood of cure may have been associated with higher coverage of the family health strategy, such as in the city’s West Zone, where PHC coverage has exceeded 90% since 2010, in addition to Rocinha with 100% coverage since 2012. Meanwhile, for the rest of the South Zone and the Tijuca neighborhood, the higher TB cure rates are likely due to easier access to other health services, better mean socioeconomic status, and retention of qualified physicians.

As expected, spatial analysis showed an association between TB cure and better socioeconomic conditions, including schooling and income [11].

There was an apparent paradox between the lower probability of cure in the elderly when analyzing the age variable at the individual level and the higher likelihood of cure with higher elderly rates at the census tract level. This may be explained by the fact that at the individual level, older patients tend to adhere less to treatment due to intrinsic factors, with more adverse effects, increased drug-drug interactions, forgetting to take medication, and lower immunity. On the other hand, patients living in census tracts with higher elderly rates have greater likelihood of cure, probably because this variable represents more structured communities in socioeconomic terms, leading to longer life expectancy, and in temporal terms because they are communities that have been settled longer and have better social support networks, favoring better treatment and thus higher likelihood of cure. Interestingly, elderly rate was the variable with the highest likelihood of cure (OR 9.39) and widest variation (95% CI 1.03–85.26), evidencing the importance of the social context for TB cure.

Several previous studies have evaluated the association between TB incidence and TB mortality and socioeconomic factors [2–4, 6, 7, 9, 41]. One study constructed a personalized social risk indicator for TB. The authors found an association between lower income, poverty, education, and overcrowding and TB mortality [7].

Household crowding is associated with higher interpersonal contact, thereby increasing the likelihood of *M. tuberculosis* transmission [3]. Uppal et al. (2021) concluded that the overall parameter representing the relative risk of progression to active disease among individuals in crowded homes compared to non-crowded homes was the most influential factor in driving costs and effectiveness [2]. Silva (2016) found that highest density of cases was strongly associated with higher population density but not with lower income or level of literacy [6]. Nevertheless, our study did not show a significant association between the number of individuals living in the household and TB cure rate.

Despite the association between race/color and cure in the bivariate analysis, no association was found in the final spatial analysis model. This race/color variable may have been subject to data entry problems. The spatial model demonstrated that the higher the educational level, the higher the likelihood of cure, which is consistent with reports in the literature [5, 40, 42].

HIV continues to be a major contributor to morbidity and mortality around the globe and remains a public health priority in Latin America [43]. Positive HIV serology and alcohol abuse were associated with lower likelihood of TB cure. This finding has been reported by other authors such as Uppal et al. (2021) [2]. Besides the expected lower immunity, these patients may show less adherence, due to drug interaction and greater occurrence of the medication’s side effects [44–47].

The Stop TB Strategy of the World Health Organization (WHO) recommends household contact investigations (HCI) for active screening of TB disease among contacts of smear- positive TB cases [1]. Saunders et al. (2018) found higher risk of progression to TB disease among close contacts of pulmonary TB cases, but the diagnostic accuracy to predict each outcome is poor [41]. Another study concluded that proactive social policies and active contact tracing to identify missed cases may help reduce the TB burden in this setting [5]. Our study found that household contact investigation was associated with higher TB cure.

It is important that governments take responsibility for ensuring universal health coverage as a key element in achieving global goals [5, 41]. In one study, primary care coverage was inversely associated with TB mortality in children [9]. Ross et al. (2018) concluded that greater population coverage of Family Health Program teams (PHC) was associated with lower TB and HIV mortality [11]. In our study, longer time between deployment of family health teams and diagnosis of the disease was associated with higher odds of TB cure, except for the category of 41 months or more, which showed the worst probability of cure among the categories. The data were insufficient to explain this phenomenon, since one would expect that patients covered by PHC for 41 months or more would be more likely to achieve cure. One possible explanation is that the first family health teams in the city of Rio de Janeiro were deployed in socioeconomically vulnerable areas, and despite efforts by the Municipal Health Department, some of these teams remained incomplete for a long time due to shortage of medical staffing.

### Strengths and limitations

Our study used comprehensive modeling based on a theoretical model relating lack of TB cure to environmental factors, access to health services, social determinants, and individual factors.

Considering that TB is closely related to socioeconomic factors, we incorporated these variables using data from Brazil’s 2010 population census. However, in some cases our results reflect limited data with imprecise measures because we applied these variables at the census level but the vital records at the individual level.

TB is closely related to housing conditions such as distance between dwellings, number of persons living in the same household, areas of social vulnerability, and sanitation, among others [3]. The spatial element is thus closely related to tuberculosis. Therefore, spatial analysis is essential in TB statistical analysis. However, most studies evaluating the spatial distribution of TB cases use data from ​polygon areas. The approach to estimation with small areas is a challenge in spatial models [11], in which the global spatial autocorrelation index should be analyzed. This can be an important barrier when the prevalence is low in the population, generating many polygons with zero cases. Therefore, it is challenging to use small polygons, closer to the cases occurred, such as polygons of the city’s census tracts, neighborhoods, or regions in a city. Another common and well-known phenomenon in these situations is the oscillation of small numbers, that is, in a small population, a random case ends up generating a high incidence rate.

Our study used a spatial analysis methodology based on geostatistics. We used the points of spatial coordinates represented by the latitude and longitude of addresses for TB cases as the units of analysis, thereby addressing the biases in spatial analysis using data aggregated in polygons.

Nevertheless, the benefits of resolving data aggregation bias in polygons should be weighed against the risk of potentially identifying individuals when analyses of exceptionally rare outcomes are conducted in extremely small areas.

It was also possible to evaluate clusters of new TB cases in the period in different areas of the city, using the Kernel point density method [22]. This information is useful for identifying areas of greatest vulnerability and population density. The use of generalized additive model (GAM) allowed identifying areas at greater risk of lack of TB cure in the city, including level of confidence and statistical significance. Such information is useful for health system administrators to prioritize areas in the city for intensifying measures to improve TB cure rates.

Finally, GAM allowed the incorporation of the time and space components into the modeling and thus the spatial analysis of PHC performance over time in TB cure. It was also possible to establish the minimum time for deployment of primary care teams to improve the results in TB cure.

The study was subject to various limitations. First, the study design does not allow establishing a causal relationship, and external validity may not be reached. Furthermore, we used data from secondary sources; the data may not be complete, introducing some bias in the study.

Second, some residential addresses were either missing, incomplete, or impossible to geocode, with 24.22% of the reported cases that could not be geocoded. The geocode loss rate in this study is worse than the rate in other studies in Brazil [6]. Silva et al. (2016) reached 94.6% geocoding in their sample of 387 cases [6].

This may be explained by the fact that the address is not validated prior to data entry, compromising the record’s quality. Furthermore, there are numerous favelas in the city of Rio de Janeiro, and there are few official records of street names and zip codes in these communities, further compromising the reports´ quality and making geocoding more difficult. Such cases probably occurred in areas with the worst socioeconomic conditions.

This may represent a classification bias, since coverage by primary care did not occur homogeneously in the city, but prioritized areas of greatest social vulnerability and may have presented greater loss of geocoding, compromising the data on primary care coverage.

Third, to avoid selection bias, we attempted to include all reported TB cases in the city during the study period. However, approximately 1600 records were not used, due to lack of information on treatment completion, which may have produced a selection bias if these cases were not randomly distributed in the city.

Finally, only 3 years of TB cases were analyzed. For the study’s results and conclusions to be more robust, a longer historical series would be necessary. This would allow assessing the impact of the consolidation effect in the primary care model on TB cure in the city of Rio de Janeiro.

### Implications and future research

Our findings are important for informing Brazilian policy and orienting further research on primary care-based TB treatment. The study allowed evaluating the relationship between PHC coverage and TB cure, based on spatial and temporal distribution. It was also possible to identify risk areas for failure to cure TB in the city of Rio de Janeiro, providing a comprehensive model of TB cure utilizing spatial and temporal components in the analysis.

TB interventions, such as active case tracing and mobile testing units can be resource-intensive and are utilized most effectively when prioritized to high-burden areas

Future studies to assess PHC performance in TB treatment should be implemented with careful consideration of how to address perceived barriers, especially studying a longer temporal dataset in the study. The results may also interest policymakers facing similar decisions in other countries.

Future work may assess whether factors such as treatment-seeking behavior and case-reporting completeness can be used to improve modelling of TB incidence from case notifications. Enhancing the quality of residential address data entry will be useful for all spatial analysis studies using geocoding, which is a huge challenge in these studies.

## Conclusion

Mapping of TB cases is a convenient tool for spatial characterization of the diseases. The results help improve our understanding of TB distribution and identification of areas at highest risk of infection. Spatial analysis of cases and temporal analysis from 2012 to 2014 showed significant associations with TB cure. In addition, areas of the city of Rio de Janeiro with odds of TB cure were identified, thus helping health system administrators implement more efficient TB control measures. Regarding coverage of primary healthcare, the study showed a significant association between TB cure and time since implementation of the family health strategy after adjusting for socioeconomic variables. This finding corroborates the importance of treating TB at the primary care level. The study can provide a template for other countries to evaluate their models of care.

## Data Availability

The datasets analyzed in this study consisted of all confirmed cases of tuberculosis, as specified in the International Classification of Diseases, 10th revision (ICD-10). Data recorded in the Information System on Diseases of Notification (SINAN) and used as the basis for the study’s results are available from the Rio de Janeiro Municipal Health Department. The data are also available from the authors themselves upon reasonable request and with permission from the Rio de Janeiro Municipal Health Department. Construction of the spatial analysis maps used the cartographic base of the city of Rio de Janeiro, available in the online database of the Brazilian Institute of Geography and Statistics (IBGE).

## Abbreviations

AIDS: Acquired immunodeficiency syndrome
API: Application Programming Interface
CI: Confidence interval
SINAN: Brazilian National Information System on Diseases of Notification
GAM: Generalized additive model
HIV: Human immunodeficiency virus
IBGE: Brazilian Institute of Geography and Statistics
OR: Odds ratio
PHC: Primary healthcare
BRL: (Brazilian reais, the official national currency)
TB: Tuberculosis
WHO: World Health Organization
